# Development of amoxicillin resistance in *Escherichia coli* after exposure to remnants of a non-related phagemid-containing *E. coli*: an exploratory study

**DOI:** 10.1186/s13756-020-00708-7

**Published:** 2020-03-16

**Authors:** Joep J. J. M. Stohr, Marjolein F. Q. Kluytmans-van den Bergh, Carlo J. M. M. Verhulst, John W. A. Rossen, Jan A. J. W. Kluytmans

**Affiliations:** 1 Department of Infection Control, Amphia Hospital, Breda, the Netherlands; 2 Laboratory for Medical Microbiology and Immunology, Elisabeth-TweeSteden Hospital, Tilburg, the Netherlands; 3 Amphia Academy Infectious Disease Foundation, Amphia Hospital, Breda, the Netherlands; 4 Julius Center for Health Sciences and Primary Care, University Medical Center Utrecht, Utrecht University, Utrecht, the Netherlands; 5 Department of Medical Microbiology and Infection Prevention, University of Groningen, University Medical Center Groningen, Groningen, the Netherlands

**Keywords:** Sterillium, AmpC, Antimicrobial resistance, Disinfection

## Abstract

**Objective:**

To determine the effect of exposure to remnants of a phagemid-containing *E. coli*, killed by treatment with a propanol-based hand rub, on antimicrobial resistance in *E. coli* isolates.

**Methods:**

An in vitro model was developed in which a clinical *E. coli* isolate (EUR1) was exposed to remnants of an *E. coli* K-12 strain containing a phagemid (pBS-E12) strain treated with Sterillium®. A series of 200 experiments was performed using this in vitro model. As a control, a series of 400 experiments was performed where the EUR1 was exposed either to the remnants of an *E. coli* K-12 strain (not containing a phagemid) (E12) treated with Sterillium® (*n* = 200) or to dried Sterillium® only (*n* = 200). The number of experiments that showed growth of an amoxicillin-resistant EUR1 isolate was evaluated in all three groups. An additional 48 experiments were performed in which a different clinical *E. coli* isolate (EUR2) was exposed to remnants of the pBS-E12 treated with Sterillium®. Whole-genome sequencing and phenotypic testing for AmpC beta-lactamase production was performed to investigate the mechanism behind this resistance development.

**Results:**

In 22 (11.0%) of 200 experiments in which the EUR1 isolate was exposed to remnants of a pBS-E12 an amoxicillin-resistant mutant isolate was obtained, as opposed to only 2 (1.0%) of 200 experiments involving the exposure of the EUR1 to Sterillium® only (risk difference: 10.0%; 95% CI 5.4–14.6%)) and 1 (0.5%) of 200 experiments involving the exposure of the EUR1 isolate to the remnants of the phagemid-free E12 (risk difference: 10.5%; 95% CI 6.1–14.9%). In 1 (2.1%) of the 48 experiments in which the EUR2 isolate was exposed to remnants of a pBS-E12 an amoxicillin-resistant mutant isolate was obtained. The development of resistance in all experiments was due to mutations in the promoter/attenuator region of the chromosomal AmpC beta-lactamase (cAmpC) gene leading to cAmpC hyperproduction.

**Conclusion:**

Exposure of an *E. coli* isolate to another phagemid-containing *E. coli* that was treated with propanol-based hand rub increased the development of amoxicillin resistance*.* Although phagemids are cloning vectors that are not present in clinical isolates, this finding may have implications for hand disinfection practices in healthcare facilities.

## Introduction

The last decades we have seen a dramatic worldwide increase in antimicrobial resistance (AMR) among Gram-negative bacteria. One of the most remarkable phenomena is the rapid increase of plasmid-mediated beta-lactam resistance in *Escherichia coli* [1–3]. International infection control guidelines recommend several measures to control the spread of AMR, among which propanol-based disinfection of hands is vital [4, 5]. These disinfection methods rapidly and effectively decrease the number of viable bacteria on hands and thereby limiting the spread of resistant bacteria and healthcare-related infections [6–9]. However, intact bacterial DNA could potentially persist after propanol-based bacterial cell lysis and mechanical cleaning and serve as a source of resistance determinants for other bacteria that reach the disinfected area [10, 11]. Uptake of plasmid and chromosomal bacterial DNA from the environment through natural transformation has already been described as a method of resistance acquisition in streptococci*, Helicobacter* spp*.* and various other bacteria [12–16]. Recent studies have also shown that *E. coli* is able to take up DNA in various environments [16–21]. However, it remains unclear to what extent the uptake of resistance plasmids via natural transformation contributes to the development of AMR in *E. coli*. Moreover, it is unknown whether exposure to environmental DNA remainders (plasmids) could facilitate this form of AMR acquisition. Therefore, we developed an in vitro model in which a clinical *E. coli* isolate (EUR1) was exposed to a pBleuscript KS(−) phagemid (encoding an amoxicillin resistance gene)-containing *E. coli* K-12 strain which had been treated with alcohol (Sterillium®). In this experiment, we did not observe plasmid transfer (results on file), yet we did observe the development of beta-lactam resistance in the EUR1 isolate. This observation led to the hypothesis that exposure to remnants of phagemid-containing *E. coli* (killed by treatment with Sterillium®) could lead to the development of AMR in *E. coli* isolates that came in contact with the remnants of the killed *E. coli* K12 strain, through increased chromosomal mutations. Although not previously reported for *E. coli,* studies have already shown that in *Salmonella* spp. and *Pseudomonas* spp. the presence of external DNA could lead to an increase in the development of AMR through mechanisms other than transformation [22, 23]. In this exploratory study, we used an in vitro model to compare the rate at which amoxicillin-resistant mutants developed for an *E. coli* isolate exposed to amoxicillin and remnants of a pBleuscript KS(−) phagemid containing *E. coli* K-12 strain treated with Sterillium®, amoxicillin and remnants of a phagemid-free K-12 strain treated with Sterillium® or amoxicillin and dried Sterillium® only.

## Method

### Isolate selection

An *E. coli* -K12 (JM83, ATCC® 35607™) harbouring a II-pBleuscript KS (−) phagemid (ATCC® 87047™) (copy number: 300–500) containing an amoxicillin-resistance gene (*bla*_TEM-116_) (pBS-E12), was chosen to be exposed to the propanol-based hand rub and two *E. coli* isolates (EUR1 and EUR2) obtained from routine clinical cultures (EUR1: peritoneal fluid culture, EUR2: urine culture) of epidemiologically unrelated patients were selected to be exposed to remnants of the pBS-E12, Sterillium® and amoxicillin (for mutant selection). Strain characteristics of the used strains are depicted in Supplementary table S1. Features of the high copy number pBleuscript phagemid are depicted in Supplementary table S2. The selection of the EUR1 and EUR2 was based on antimicrobial susceptibility pattern, i.e. susceptible to amoxicillin and resistant to trimethoprim based on EUCAST clinical breakpoints version 9.0 [24]. The pBS-E12 has a trimethoprim MIC below the clinical breakpoint. Antimicrobial susceptibility testing was performed using the VITEK 2® system (bioMérieux, Marcy l’Etoile, France). The minimal inhibitory concentration (MIC) for amoxicillin was additionally tested with ETEST® (bioMérieux, Marcy l’Etoile, France). The MICs of the EUR1, EUR2, and pBS-E12 for the various tested antimicrobial agents are shown in Table 1.

**Table 1:** Minimal inhibitory concentration for various antibiotics

Isolate	MIC (mg/L)							D68C AmpC & ESBL Detection set conclusion
	E-test®	Vitek®						
	amox	amcl	pita	cfrx	cfxt	cftz	cftx	AmpC hyperproduction¥
pBS-E12	> = 256	> = 32	> = 128	8	<=4	<=1	<=1	No
EUR1	6	<=2	<=4	4	<=4	<=1	<=1	Yes
EUR1M1	> = 256	> = 32	8	32	> = 64	4	<=1	Yes
EUR1M2	> = 256	> = 32	16	32	> = 64	4	<=1	Yes
EUR1M3	> = 256	> = 32	<=4	16	16	<=1	<=1	Yes
EUR1M4	> = 256	> = 32	8	32	32	<=1	<=1	Yes
EUR1M5	> = 256	> = 32	8	> = 64	> = 64	4	2	Yes
EUR1M6	256	> = 32	<=4	16	> = 64	<=1	<=1	Yes
EUR1M7	> = 256	> = 32	8	32	> = 64	4	<=1	Yes
EUR1M8	> = 256	> = 32	<=4	16	16	<=1	<=1	Yes
EUR1M9	256	> = 32	<=4	16	16	<=1	<=1	Yes
EUR1M10	128	> = 32	<=4	16	32	<=1	<=1	Yes
EUR1M11	128	> = 32	<=4	32	16	<=0.25	<=0.25	Yes
EUR1M12	> = 256	> = 32	16	32	16	2	1	Yes
EUR1M13	> = 256	> = 32	16	32	32	2	1	Yes
EUR1M14	> = 256	> = 32	16	32	16	1	<=0.25	Yes
EUR1M15	196	> = 32	<=4	16	16	0.5	<=0.25	Yes
EUR1M16	196	> = 32	<=4	16	16	0.5	<=0.25	Yes
EUR1M17	256	> = 32	<=4	32	32	2	0.5	Yes
EUR1M18	> = 256	> = 32	16	32	16	2	1	Yes
EUR1M19	> = 256	> = 32	8	32	16	2	<=0.25	Yes
EUR1M20	64	16	<=4	4	<=4	0.5	<=0.25	Yes
EUR1M21	> = 256	> = 32	16	32	16	4	1	Yes
EUR1M22	256	> = 32	16	32	16	4	1	Yes
EUR1S1	> = 256	> = 32	<=4	16	32	<=1	<=1	Yes
EUR1S2	> = 256	> = 32	8	32	> = 64	2	<=1	Yes
EUR1E1	> = 256	> = 32	<=4	16	32	<=1	<=1	Yes
EUR2	1	<=2	<=4	2	<=4	<=1	<=1	No
EUR2M1	> = 256	> = 32	8	8	<=4	<=1	<=1	Yes

MICs were measured with ETEST® (bioMérieux, Marcy l’Etoile, France) (amoxicillin) or the VITEK 2® system (bioMérieux, Marcy l’Etoile, France). ¥Interpretation of D68C AmpC & ESBL Detection set according to manufacturers instruction. Zone diameters as measured in the D68C AmpC & ESBL Detection set are depicted in Supplementary table S3. amox: amoxicillin; amcl: amoxicillin-clavulanic acid; pita: piperacillin-tazobactam; cfrx: cefuroxime; cfxt: cefoxitin; cftz: ceftazidime; cftx: cefotaxime; mero: meropenem; imip: imipenem;M: amoxicillin-resistant mutant after exposure to remnants of the pBS-E12 and Sterillium®S: amoxicillin-resistant mutant after exposure to Sterillium® onlyE: amoxicillin-resistant mutant after exposure to E12 and Sterillium®

### Exposure to propanol-based hand rub (Sterillium®) and resistance induction

**Fig. 1:**
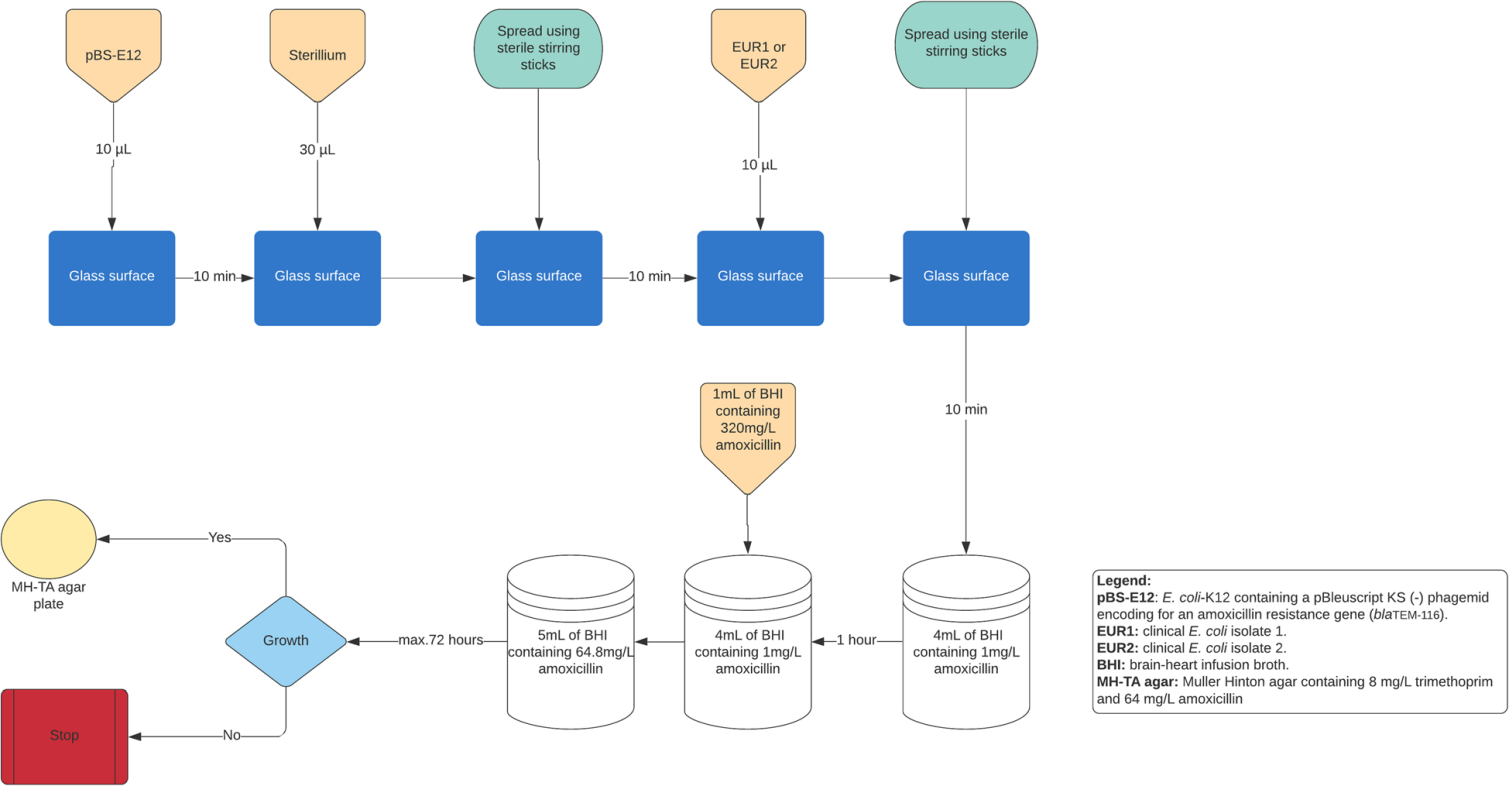
An illustration of the experiments in which the EUR1 or EUR2 isolates were exposed to amoxicillin and the pBS-E12 treated with Sterillium®

Sterile glass surfaces (25mmx18mm) were inoculated with 10 μL of a 0.5 McFarland suspension of the pBS-E12 isolate (1.5 × 10^6^ colony forming units (CFU)) in brain-heart infusion broth (BHI). The inoculation of the glasses was performed in a Kojair biosafety cabinet class II Silver Line. The inoculated glasses were left to dry at ambient air temperature for 10 +/− 1 min. Subsequently, the glasses were inoculated with 30 μL of Sterillium®, which was spread across the entire glass surface using sterile plastic sticks. The Sterillium® used contained per 100 g of solution: Propan-2-ol 45.0 g, propan-1-ol 30.0 g and mecetronium etilsulfate 0.2 g. The ratio of the volume of inoculated Sterillium® to glass surface area (3 × 10 ^− 5^ L: 0.00045 m^2^) was chosen to reflect the ratio of the volume of Sterillium® used in hand disinfection to the average hand surface area (3 × 10^− 3^ L: 0.045 m^2^) [25, 26]. The PBS-E12 and Sterillium®-containing glasses were left to dry at ambient room temperature for 10 +/− 1 min. Subsequently, the glasses were inoculated with 10 μL of an 0.5 McFarland suspension of the EUR1 isolate (1.5 × 10^6^ CFU) in BHI, which was spread across the entire glass surface using sterile plastic sticks. The re-inoculated glasses were left to dry at ambient air temperature for 10 +/− 1 min. Subsequently, the glasses were placed in a container with 4 mL of BHI, containing 1 mg/L amoxicillin, vortexed for 30 s at 2800 rotations per minute and incubated at 35 to 37C°. The duration the isolates were subjected to a subinhibitory amoxicillin concentration is meant to represent the duration of subinhibitory amoxicillin plasma-concentrations in patients starting amoxicillin treatment [27]. After 1 h of incubation, an additional 1 mL of BHI broth containing 320 mg/L amoxicillin was added to the glasses carrying containers, resulting in 5 mL of BHI broth containing 64.8 mg/L amoxicillin in the glasses carrying containers. The containers were then incubated at 35 to 37C° for 72 h and visually inspected for bacterial growth at 8, 24, 48 and 72 h. At visible growth or after 72 h of incubation in absence of visible growth, 1 μL of the suspension was plated on a Muller Hinton agar containing 8 mg/L trimethoprim and 64 mg/L amoxicillin (MH-TA). The inoculated MH-TA plates were incubated for 18 to 24 h at 35 to 37C°. Colonies growing on the MH-TA underwent species identification performed with VITEK MS (bioMérieux, Marcy l’Etoile, France), using the VITEK MS Knowledge Base v.2.0, and antimicrobial susceptibility testing performed with the VITEK 2® system (bioMérieux, Marcy l’Etoile, France), using EUCAST susceptibility breakpoints version 9.1 [24]. The MIC of all isolates to amoxicillin was additionally tested using ETEST® (bioMérieux, Marcy l’Etoile, France). Grown *E. coli* colonies with a MIC of > 8 mg/L for trimethoprim, measured in the VITEK 2® system (bioMérieux, Marcy l’Etoile, France), and with a MIC of > 64 mg/L for amoxicillin, measured with ETEST® (bioMérieux, Marcy l’Etoile, France), were considered mutants. A series of 200 experiments were performed using the EUR1 isolate. Additionally, a series of 48 experiments were performed using the EUR2 isolate. Experiments with the EUR1 and EUR2 isolates were performed on separate days. For each experiment colonies were picked of the EUR1 and EUR2 isolate and the pBS-E12 strain in stationary growth phase after incubation at 35 to 37C° for 18 to 24 h on Muller Hinton agar. The pBS-E12 strain was inoculated on Muller Hinton agar plates containing an amoxicillin 10 μg disk. Only colonies of the pBS-E12 growing in the direct surrounding of the amoxicillin disk were used in the experiments. The experimental setup is illustrated in Fig. 1.

### Control experiments

**Fig. 2:**
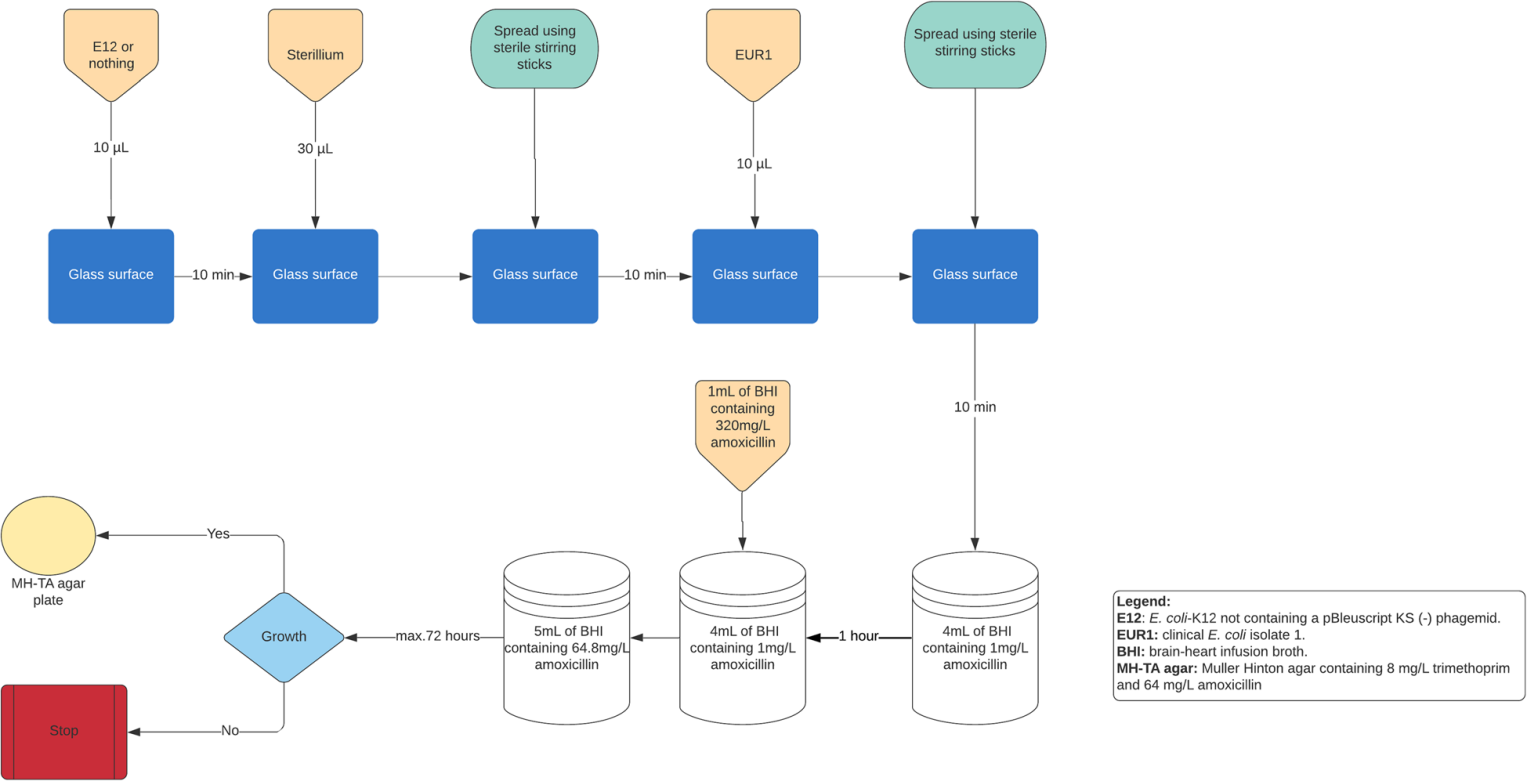
An illustration of the experiments in which the EUR1 isolate was exposed to amoxicillin and the E12 treated with Sterillium®, or to amoxicillin and Sterillium®

A series of 200 control experiments were performed in which the glasses were only inoculated with Sterillium® and the EUR1 and a series of 200 experiments were performed in which the glasses were inoculated with the *E. coli* K-12 strain (JM109, ATCC® 53323™) not containing the pBleuscript KS (−) high copy number phagemid (E12) (Supplementary Table S1), Sterillium® and the EUR1 isolate. Experiments were performed on separate days. For each experiment colonies were picked of the EUR1 isolate and E12 strain after incubation at 35 to 37C° for 18 to 24 h on Muller Hinton agar. The setup of the control experiments is illustrated in Fig. 2.

### Disinfection experiments

To determine if the pBS-E12 was still culturable after exposure to Sterillium®, we performed a series of 100 experiments in which the glasses were inoculated with the pBS-E12 followed by Sterillium® only. The inoculated glasses were placed in a container with 5 mL of BHI, vortexed for 30 s at 2800 rotations per minute and incubated for 72 h at 35 to 37C°, visually inspecting growth at 24, 48 and 72 h. In case there was no visible growth after 72 h of incubation, 1 μL of broth was plated on Muller Hinton agar.

### Whole-genome shotgun sequencing (WGS) and de novo assembly

The wildtype isolates (EUR1, EUR2), the pBS-E12 and E12 strains, and a selection of the mutant isolates were sequenced on an Illumina MiSeq (Illumina, San Diego, United States) and assembled with CLC Genomics Workbench v.11 or v.12 (Qiagen, Hilden, Germany). Selection criteria were: in the first thirteen experiments in which growth of a mutant isolate was detected, an isolate was sent for sequencing. The following quality control criteria were used: coverage ≥20; number of scaffolds ≤1000; N50 ≥ 15.000 bases and maximum scaffold length ≥ 50.000 bases.

### Mechanism of resistance

The presence of genomic resistance determinants conferring amoxicillin and trimethoprim resistance in the assembled genomes of the mutated *E. coli* was identified with the online bioinformatic tools ResFinder v3.1 and PointFinder v3.1 (Center for Genomic Epidemiology, Technical University of Denmark, Lingby, Denmark) [28, 29]. Genomes were screened for known and unknown chromosomal resistance mutations. Acquired resistance genes were called when at least 60% of the length of the best matching gene in the ResFinder database was covered with a sequence identity of at least 90%. If an unknown or known chromosomal mutation was detected in genomic regions implicated in beta-lactam resistance using PointFinder, the region was extracted from the assembled genome using Biopython v.1.73. Subsequently, the extracted genomic regions were aligned using Vector NTI Advance 11 software (ThermoFisher Scientific, Waltham, USA) to the corresponding region of either the EUR1 or EUR2 isolate and the corresponding region of the *E. coli* K-12 strain MG1655 (GenBank database accession number NC000913.3), the pBS-E12 strain, and the E12 strain. Moreover, to verify that the observed amoxicillin resistance was not the result of the acquisition of the amoxicillin resistance gene from the pBleuscript(−) phagemid, all assembled genomes were screened for the presence of this gene using ABRicate v.0.8.13 with the same coverage and identity thresholds as those used in ResFinder. Additionally, all mutated strains and the pBS-E12, EUR1, EUR2 isolates were phenotypically screened for AmpC production using the D68C AmpC & ESBL Detection set (Mastdiscs, Mastgroup Ltd., Bootle United Kingdom) and interpreted according to manufacturer’s instruction.

### Whole-genome multilocus sequence typing

Whole-genome multilocus sequence typing (wgMLST) (core and accessory genome) was performed of both wildtype isolates and all sequenced mutant isolates using Ridom SeqSphere+, version 5.1.0. (Ridom, Münster, Germany). Species-specific typing schemes were used as described by Kluytmans-Van den Bergh et al. [30]. All-to-all pairwise genetic difference was calculated between the isolates by counting the total number of allele differences in the wgMLST typing scheme and by dividing the total number of allele differences in the wgMLST typing scheme by the total number of shared alleles in the wgMLST typing scheme, ignoring pairwise missing values.

### Statistics

Risk differences were estimated using a generalised linear model with binomial distribution, an identity link and robust error estimation (SPSS version 25).

### Accession numbers

All generated raw reads were submitted to the European Nucleotide Archive (ENA) of the European Bioinformatics Institute (EBI) under the study accession number: PRJEB34354.

## Results

### Resistance induction experiment

In 22 (11.0%) of 200 experiments in which the EUR1 strain was exposed to the remnants of the pBS-E12 and Sterillium® an amoxicillin- and trimethoprim-resistant *E. coli* isolate (EUR1M1 - EUR1M22) was obtained*,* as opposed to only 2 (1.0%) (EUR1S1, EUR1S2) of 200 experiments involving the exposure of the EUR1 strain to Sterillium® only (risk difference 10.0%; 95% CI 5.4–14.6%), and 1 (0.5%) (EUR1E1) of 200 experiments involving the exposure of the EUR1 strain to the remnants of the E12 and Sterillium® (risk difference 10.5%; 95% CI 6.1–14.9%). In the experiments performed using the EUR2, 1 mutant isolate (EUR2M1) was grown in 48 experiments. Amoxicillin-resistant strains did not only show increased MICs for amoxicillin but also for amoxicillin-clavulanic acid, piperacillin-tazobactam or cephalosporins (Table 1). No increases in the MIC were observed for carbapenems and non-beta-lactam antibiotics tested. One out of 100 disinfection experiments showed growth of the pBS-E12, indicating that in only a minimal number of experiments the amoxicillin concentration during the experiments was influenced by viable beta-lactamase-producing pBS-E12.

### Mechanisms of amoxicillin resistance

A selection of the isolates was sequenced, i.e. 10 mutant *E. coli* isolates from the experiment in which EUR1 was exposed to the remnants of the pBS-E12, 2 mutants from the control experiments, 1 mutant from the experiments with the EUR2 isolate, the wildtype isolates EUR1 and EUR2, and the pBS-E12 and E12 strains. Table 2 shows the various mutations at given positions of the promoter/attenuator region of the cAmpC gene for every sequenced isolate in this study. Known and unknown mutations in the promoter/attenuator region of the chromosomal AmpC beta-lactamase (cAmpC) gene were detected in the genomes of all mutant *E. coli* isolates that were sequenced (EUR1M1-EUR1M10, EUR1S1-EUR1S2, EUR2M1), but not in the EUR1 isolate. Two unknown mutations were detected in the promoter/attenuator region of the EUR2 isolate (Table 2). Alignment against the corresponding region of an *E. coli* K-12 strain revealed that one of these mutations was in the alternate − 10 box promoter box (Table 2). However, these mutations did not increase the MIC for amoxicillin in the EUR2 isolate. In every mutant (EUR1M1-EUR1M10, EUR1S1-EUR1S2, EUR2M1) alignment of the cAmpC promoter/attenuator region revealed mutations, when compared to the same genomic region of the corresponding wildtype isolates, in regions implicated in cAmpC hyperproduction as described by Tracz et al. (Table 2) [31]. Moreover, the different promoter/attenuator regions present in the mutant EUR1 isolates (Table 2) suggests de novo mutation rather than the selection of a previously present mutated subpopulation. No mutations in other regions implicated in beta-lactam resistance were detected, nor were acquired beta-lactam resistance genes detected in any of the mutated (EUR1M1-EUR1M10, EUR1S1-EUR1S2, EUR2M1) or wildtype isolates (EUR1, EUR2). The amoxicillin-resistance gene of the pBleuscript KS(−) phagemid was not detected in any of the sequenced isolates except the pBS-E12. In the pBS-E12 and E12 strain alignment of the cAmpC promotor/attenuator did not reveal mutations, when compared to the same genomic region of the *E. coli* K-12 strain MG1655. A dfrA1 gene conferring trimethoprim resistance was detected in the EUR1, EUR2 and all sequenced mutant isolates but not in the pBS-E12 and E12 strains.

**Table 2:** Mutations in cAmpC promoter/attenuator region of the wildtype and mutant isolates

*E.coli* K-12 (wildtype)	Base in wildtype cAmpC promoter/attenuator region									
	−32	− 23.1	− 21.1	−19	− 18	−16.1	− 11	+ 58	+ 81	
	T	–	–	T	G	–	C	C	G	
Isolate	Position of mutation in cAmpC promoter/attenuator region^a^									Date experiments performed^¤^
	−32	−23.1	−21.1	−19	−18	−16.1	−11	+ 58	+ 81	
pBS-E12^¥^										
E12										
EUR1 (wildtype)									A	
EUR1M1	A								A	10th May
EUR1M2		G	C	A					A	10th May
EUR1M3		G	C	A					A	11th May
EUR1M4		G	C	A					A	12th May
EUR1M5		G	C	A					A	12th May
EUR1M6							T		A	13th May
EUR1M7		G	C	A					A	25th May
EUR1M8		G	C	A					A	26th May
EUR1M9		G	C	A					A	1st June
EUR1M10							T		A	1st June
EUR1S1			T						A	23th May
EUR1S2	A								A	17th August
EUR2 (wildtype)					A			T		
EUR2M1					A	A		T		12th May

^a^Position numbering of the cAmpC promoter/attenuator region as defined by Mulvey et al. [31]. In position number n.x: decimal number x refers to an insertion at position n. Positions −32 and − 11 are part of the wild-type promoter boxes. Position − 19 is part of the alternate promoter box. Positions − 21.1 and − 23.1 are part of the spacer region of both the wild-type and alternate promoter. Position − 16.1 is part of the spacer region of the wild-type promoter. ¥ strain used in experiments on 1st of June. ¤ all experiments were performed in 2016

Phenotypic tests for AmpC beta-lactamase production were negative for the two wildtype isolates and the pBS-E12 isolate. However, for all mutants, phenotypic testing showed AmpC beta-lactamase production (Table 1**;** Supplementary Table S3).

### Whole-genome multilocus sequence typing

**Fig. 3:**
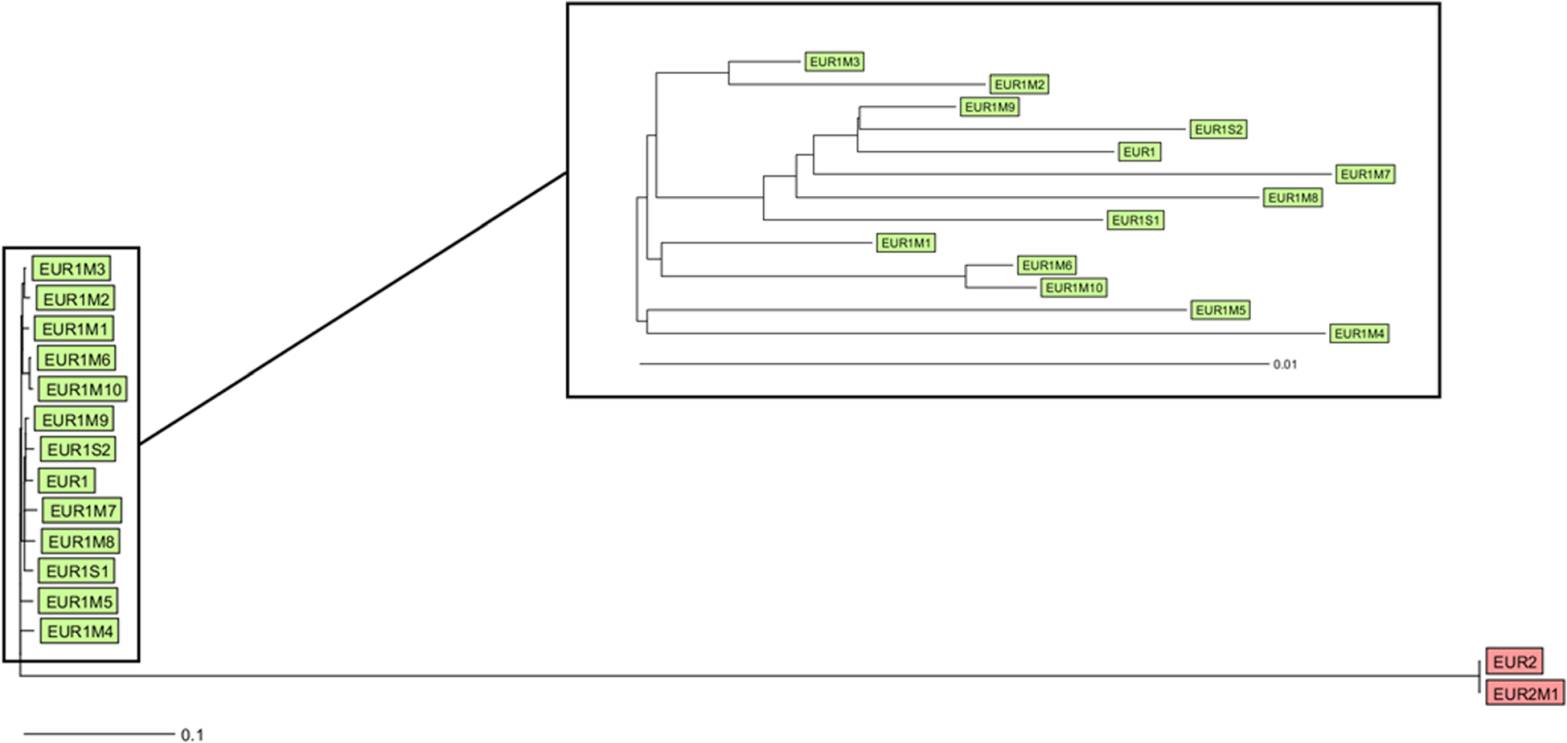
A Neighbour-joining tree representing the percentage of wgMLST allele differences between all sequenced isolates (Green: EUR1 wildtype/mutant isolates; Red: EUR2 wildtype/mutant isolates)

The number of allele differences between the mutated EUR1M1-EUR1M10 isolates and wildtype EUR1 isolate ranged from 21 (0.59%) to 69 (1.96%) (median: 44.5 (1.27%)). In all but one (EUR1M9) of the mutant EUR1 isolates, the difference between the mutant and corresponding control exceeded the threshold for genetic distance between related and unrelated isolates as defined by Klutymans-van den Bergh et al. (Fig. 3) [30]. Both mutated isolates from the control experiment (EUR1S1 and EUR1S2) also showed a high number of allele differences when compared to the EUR1 wildtype (EUR1S1: *n* = 34 (0.96%), EUR1S2: *n* = 36 (1.02%)). The high number of allele differences between the various mutated EUR1 isolates suggests de novo mutation acquisition rather than the selection of a previously present mutated subpopulation (Fig. 3). In the EUR2M1, when compared to its wildtype, no allele differences were observed. The number of allele differences between the EUR1 and EUR2 isolate was 3184 (97.73%)(Fig. 3).

## Discussion

An in vitro model was developed to simulate propanol-based hand disinfection as is common practice in healthcare facilities nowadays. Bacteria are effectively killed, but their remnants remain present after disinfection. Subsequently susceptible bacteria are exposed to the remaining debris, including DNA. Experiments performed using this model showed a significant increase in the development of amoxicillin resistance in *E. coli* isolates after exposure to the remnants of the pBS-E12 treated with a propanol-based hand rub as compared to exposure to remnants of the E-12 treated with a propanol-based hand rub and as compared to exposure to only a propanol-based hand rub. AmpC beta-lactamase hyperproduction, due to mutations in the AmpC promoter/attenuator region, was responsible for the development of the observed AMR. The number of resistant mutants that developed in the isolates exposed to the remnants of the pBS-E12 also exceeded the number of pBS-E12 growing in the disinfection experiments, which indicates that amoxicillin degradation by the pBS-E12 beta-lactamase was not responsible for the observed increase in AMR. All mutated EUR1 isolates showed a high number of allele differences when compared to the wildtype isolate, suggesting genome-wide mutations. Interestingly this genome-wide mutational pattern was not detected in the EUR2 isolate. Indicating that the genome-wide mutational pattern might not only be (control) experiment but also isolate related.

The exact underlying mechanism leading to the AMR conferring mutations in the promoter/attenuator region of the cAmpC remains unknown. Since the amoxicillin resistance gene of the pBluescript phagemid was not detected in the EUR1 and EUR2 mutant isolates, it seems more likely that the remnants of the pBS-E12 increased the mutation rate in the EUR1 and EUR2 isolate facilitating AMR development through cAmpC hyperproduction. Previous studies have implicated several mechanisms that may increase the mutation rate and result in AMR, such as the general stress response and an increased constitutive mutation frequency [32–35]. Further studies are needed to evaluate the role of these mechanisms in our experiments. Moreover, mutagenic assays with other antibiotics (e.g. streptomycin and nalidixic acid) need to be performed, to further assess the mutagenic potential of the remnants of the pBS-E12 and Sterillium® in the EUR1 isolate. However, the remnants of the pBS-E12 could also facilitate the development of AMR by enabling the EUR1 and EUR2 isolates to survive longer under high amoxicillin concentrations, possibly through providing nutrients or through efflux pump activation, thereby increasing the chance of the occurrence of a mutation leading to cAmpC hyperproduction. A small degree of heterogeneity in the cAmpC promotor region of the pBS-E12 population that lead to cAmpC hyperproduction cannot be completely ruled out. Therefore, it still remains possible that the cAmpC promotor region of this heterogeneous subpopulation was horizontally transferred to the wildtype EUR1 and EUR2 isolates.

Several studies have shown that environmental stressors other than antibiotics can influence the development of AMR [35–38]. However, the association between exposure to remnants of phagemid-carrying *E. coli* isolates and increased development of AMR has not been investigated to date. Interestingly, this increase in the development of AMR was only related to exposure to remnants of phagemid-containing *E. coli* isolates. Other studies have already shown that extracellular DNA can lead to the development of AMR in other bacteria [22, 23]. However, this is the first study relating exposure to external DNA to the development of beta-lactam resistance through chromosomal mutations in *E. coli*.

Despite the increased development of AMR was only related to exposure to remnants of the pBS-E12, it remains unknown if and what specific compounds of Sterillium® contribute to the observed increased development of AMR. Contrary to other alcohol-based hand rubs, Sterillium® contains mecetronium etilsulfate which potentially has a lasting antimicrobial effect [39, 40]. Further studies are needed to evaluate if this increased development of AMR also occurs with other alcohol-based hand rubs not containing such compounds. Furthermore, the current study only simulates hand disinfection procedures so it remains unknown to what extent this phenomenon also could apply to environmental disinfection.

Although cAmpC hyperproduction has been implicated in beta-lactam resistance in *E. coli* isolates following exposure to stepwise increasing concentrations of amoxicillin over several days [41, 42]. cAmpC hyperproduction in this study occurred after only a short time of exposure to amoxicillin concentrations lower than the MIC of the exposed *E. coli* isolate. Even in our control experiments, in which the *E. coli* isolates were only exposed to Sterillium®, cAmpC hyperproduction developed after only short sub-inhibitory amoxicillin concentrations. Moreover, in the isolates EUR1M2-EUR1M5 and EUR1M7–9 three mutations were detected in the cAmpC promotor region. Each of these mutations individually could increase cAmpC production in *E. coli* [31, 43]. Perhaps, consecutive mutations, not a single mutational event during the short time of sub-inhibitory amoxicillin concentration, lead to a step-wise increase in amoxicillin MIC in these mutant isolates. In the study by Kohanski et al. [44] exposure to sub-lethal levels of amoxicillin also resulted in MIC increases to antimicrobials other than from the beta-lactam group. We did not observe MIC increases or known mutations in resistance-associated genes of antibiotics other than the beta-lactams.

Contrary to other studies investigating the development of AMR in *E. coli* [41, 44], we used two clinical *E. coli* isolates for amoxicillin resistance induction. Moreover, our in vitro model simulates propanol-based hand disinfection procedures which are very common in clinical practice [4, 5, 45]. Also, the duration of exposure to sub-inhibitory amoxicillin concentrations closely reflects the duration of sub-inhibitory amoxicillin plasma concentrations in patients at the start of amoxicillin treatment [27].

This study has some limitations. The pBluescript-KS (−) phagemid is a cloning vector not present in clinical isolates. Whether remnants of *E. coli* isolates containing wildtype phagemids also increase the development of AMR needs further investigation. Moreover, since the pBluescript KS(−) phagemid contains both a bacteriophage origin of replication and a plasmid origin of replication future studies are required to assess whether the observed effect is bacteriophage or plasmid related. Also, only a limited number of isolates were used in this study. The extent to which this AMR induction is possible in other *E. coli* isolates or other species remains unknown. Moreover, it remains unknown whether and to what extent this in vitro phenomenon plays a role in the development of AMR in vivo.

To the best of our knowledge, this is the first study showing development of amoxicillin resistance in an *E. coli* isolate after exposure to a phagemid-containing *E. coli* treated with a propanol-based hand rub.

## Conclusion

This exploratory study showed the development of amoxicillin resistance in an *E. coli* isolate after exposure to an unrelated phagemid-containing *E. coli* treated with a propanol-based hand rub. Although phagemids are cloning vectors that are not present in clinical isolates, this finding may have implications for hand disinfection practices in healthcare facilities.

## Supplementary information

**Additional file 1:Table S1.** Characteristics of used strains. **Table S2.** Features of pBleuscript KS(-) phagmid ATCC® 87047TM. **Table S3.** Inhibition zone diameters of D68C AmpC & ESBL detection set for the pBS-E12, EUR1, EUR2 and mutant isolates.

## Data Availability

The datasets supporting the conclusions of this article is included within the article and its additional file.

## Abbreviations

AMR: Antimicrobial resistance
pBS-E12: *E. coli* -K12 containing a pBleuscript KS (−) phagemid encoding for an amoxicillin resistance gene (*bla*_TEM-116_)
EUR1: Clinical *E. coli* isolate 1
EUR2: Clinical *E. coli* isolate 2
MIC: Minimal inhibitory concentration
BHI: Brain-heart infusion broth
MH-TA: Muller Hinton agar containing 8 mg/L trimethoprim and 64 mg/L amoxicillin
WGS: Whole genome shotgun sequencing
ENA: European Nucleotide Archive
EBI: European Bioinformatics Institute
cAmpC: chromosomal AmpC beta-lactamase
