# Supplementary material for: Development of amoxicillin resistance in Escherichia coli after exposure to remnants of a non-related phagemid-containing E. coli: an exploratory study
Source: Antimicrob Resist Infect Control. 2020 Mar 16;9:48. doi: 10.1186/s13756-020-00708-7 (PMC7077161; doi:10.1186/s13756-020-00708-7)
Supplement: Supplementary file 1 — Additional file 1:Table S1. Characteristics of used strains. Table S2. Features of pBleuscript KS(-) phagmid ATCC® 87047TM. Table S3. Inhibition zone diameters of D68C AmpC & ESBL detection set for the pBS-E12, EUR1, EUR2 and mutant isolates. [file 13756_2020_708_MOESM1_ESM.docx]

**Supplementary table S1. Characteristics of used strains.**

| **Strain** | **Genotype** | **Storage conditions** | **Growth conditions** | **Reference/Origin** |
| --- | --- | --- | --- | --- |
| **JM83** | **ara, Δ(lac-proAB), rspL(+strA), ϕ80, lacZΔM15.** | **-80C◦ Microbank^TM^** | **Aerobic, 35-37C°** | **ATCC^®^ 35607^TM^** |
| **JM109** | **recA1, endA1, gyrA96, thi, hsdR17, supE44, relA1, λ-, Δ(lac-proAB), [F' traD36, proAB, lacIqZΔM15].** | **-80C◦ Microbank^TM^** | **Aerobic, 35-37C°** | **ATCC^®^ 53323^TM^** |

**Supplementary table S2. Features of pBleuscript KS(-) phagmid ATCC® 87047^TM^.**

| **Feature** | **Nucleotide position** |
| --- | --- |
| **F1 origin of ss-DNA replication** | **21-347** |
| **pUC origin of replication** | **1158-1825** |
| **Amoxicillin resistance open reading frame** | **1976-2833** |

**Supplementary table S3.** Inhibition zone diameters of D68C AmpC & ESBL detection set for the pBS-E12, EUR1, EUR2 and mutant isolates.

| **Isolate** | **Inhibition zone diameter (mm)** | | | |
| --- | --- | --- | --- | --- |
|  | **D68C AmpC & ESBL Detection set** | | | |
|  | **cfpd** | **cfpd-clox** | **cfpd-clv** | **cfpd-clox-clv** |
| pBS-E12 | 27 | 27 | 27 | 27 |
| EUR1 | 28 | 29 | 29 | 29 |
| EUR1M1 | 8 | 25 | 8 | 25 |
| EUR1M2 | 8 | 24 | 8 | 24 |
| EUR1M3 | 14 | 24 | 15 | 24 |
| EUR1M4 | 9 | 19 | 9 | 19 |
| EUR1M5 | 7 | 18 | 7 | 18 |
| EUR1M6 | 12 | 27 | 12 | 27 |
| EUR1M7 | 11 | 24 | 11 | 24 |
| EUR1M8 | 14 | 25 | 14 | 25 |
| EUR1M9 | 14 | 25 | 14 | 25 |
| EUR1M10 | 14 | 27 | 14 | 27 |
| EUR1M11 | 13 | 23 | 13 | 24 |
| EUR1M12 | 13 | 25 | 13 | 25 |
| EUR1M13 | 8 | 23 | 8 | 23 |
| EUR1M14 | 12 | 23 | 12 | 23 |
| EUR1M15 | 14 | 24 | 14 | 24 |
| EUR1M16 | 14 | 24 | 14 | 24 |
| EUR1M17 | 11 | 25 | 11 | 25 |
| EUR1M18 | 11 | 25 | 12 | 25 |
| EUR1M19 | 14 | 24 | 14 | 24 |
| EUR1M20 | 19 | 28 | 20 | 28 |
| EUR1M21 | 9 | 23 | 9 | 24 |
| EUR1M22 | 11 | 22 | 11 | 22 |
| EUR1S1 | 11 | 26 | 12 | 26 |
| EUR1S2 | 9 | 24 | 9 | 24 |
| EUR1E1 | 10 | 23 | 10 | 23 |
| EUR2 | 27 | 28 | 28 | 28 |
| EUR2M1 | 14 | 25 | 14 | 25 |
| cfpd: cefpodoxim; cfpd-clox: cefpodoxim-cloxacillin; cfpd-clv: cefpodoxim-clavulanic acid; cfpd-clox-clv: cefpodoxim-cloxacillin-clavulanic acid. | | | | |
